# Alleviating Effects of Three Heat-Inactivated *Enterococcus faecalis* Strains Against Growth Suppression, Oxidative Stress and Gut Microbiome Dysbiosis in *Macrobrachium rosenbergii* Fed with Sesame Meal-Replaced Fish Meal Diet

**DOI:** 10.3390/antiox15020210

**Published:** 2026-02-05

**Authors:** Xiu Fang, Ling Zhu, Xuwen Bing, Zhengzhong Li, Xin Liu, Bo Liu, Cunxin Sun, Xiaochuan Zheng, Bo Liu

**Affiliations:** 1 College of Fisheries and Life Science, Shanghai Ocean University, Shanghai 201306, China; m230100339@st.shou.edu.cn; 2 Key Laboratory of Freshwater Fisheries and Germplasm Resources Utilization, Ministry of Agriculture and Rural Affairs, Freshwater Fisheries Research Center, Chinese Academy of Fishery Sciences, Wuxi 214081, China; zhuling@ffrc.cn (L.Z.); bingxw@ffrc.cn (X.B.); liubo96@ffrc.cn (B.L.); suncx@ffrc.cn (C.S.); 3 Wuxi Fisheries College, Nanjing Agricultural University, Wuxi 214128, China; lizz@stu.njau.edu.cn (Z.L.); 2023213007@stu.njau.edu.cn (X.L.)

**Keywords:** *Enterococcus faecalis*, postbiotics, antioxidant, intestinal microbiota, metabolomics

## Abstract

This study evaluated the alleviating effects of three heat-inactivated *Enterococcus faecalis* strains on growth suppression, oxidative stress, and gut microbiome dysbiosis in *Macrobrachium rosenbergii*-fed sesame meal-substituted fish meal diets. The trial comprised a control group (CT), low fish meal group (LF), and LF fed with three postbiotic-supplemented groups (LF+HK-448, LF+HK-798, LF+HK-804). Results demonstrated that compared with the CT group, the LF diet significantly decreased weight gain rate, specific growth rate, hepatopancreatic total nitric oxide synthase and inducible nitric oxide synthase, while increased feed conversion ratio, nitric oxide, and malondialdehyde contents. Among the postbiotics, LF+HK-804 group conferred the most pronounced compensatory growth and significantly improved oxidative stress and immune markers, as evidenced by elevated WGR, SGR, HSI, and flesh percentage, reduced MDA, and the down-regulation of *Toll* and *Relish* alongside the upregulation of *peroxiredoxin-5*. Intestinal microbiota analysis showed the group of LF+HK-804 improved microbial diversity and richness, specifically by increasing Firmicutes and decreasing *Habeamium* and *Sphingomonas*. Metabolomics identified 11 key differential metabolites related to amino acid, energy, and fatty acid metabolism. Correlation analysis further revealed that *Gemmatimonadetes*, *WD2101_soil_group*, and *Sphingomonas* were negatively correlated with phospholipids and positively correlated with glycoside and fatty acid metabolites. Moreover, immunometabolic correlation analysis segregated the metabolic response of microbiota into two distinct profiles: one potentiating a reactive oxygen/nitrogen species–antioxidant defense, and the other favoring a Dorsal/Relish-mediated transcriptional response. In conclusion, *E. faecalis* 804 can promote growth, alleviate oxidative damage, enhance immunity, and regulate intestinal microbiota and metabolic capacity in *M. rosenbergii*, showing great potential as a postbiotic.

## 1. Introduction

In the context of the rapid development of global aquaculture, the contradiction between the supply and demand of fish meal has become increasingly prominent [1]. This scarcity of fish meal has prompted the exploration of plant protein alternatives in the feed industry [2]. Among them, the sesame meal has gradually become a research hotspot due to its high methionine content and balanced amino acid composition [3]. However, the anti-nutritional factors such as phytic acid and oxalic acid in sesame meal are likely to inhibit mineral absorption and reduce the overall feed digestibility [4,5]. Therefore, it is urgent to screen out feed additives that can effectively alleviate the negative effects of the substitution of sesame meal for fish meal.

*Macrobrachium rosenbergii* is an important freshwater aquaculture species in China, with an annual output exceeding 220,000 tons. The optimal dietary crude protein level for this species is 38–40% in formulated feeds, which is primarily met by fish meal [6]. Compared with fish meal, the presence of anti-nutritional factors and inferior palatability in plant protein sources adversely affects the growth performance, nutrient digestibility, and overall health of aquatic animals [7,8]. For example, the addition level of 25% fermented soybean meal instead of fish meal can reduce the growth performance of *Penaeus vannamei*, and a more than 50% replacement level will lead to immune stress [9,10].

However, a study has also found that compared with the low fish meal group, the addition of a superior microbial combination containing *Enterococcus faecalis* can improve the survival rate of *Litopenaeus vannamei* and regulate its intestinal microbiota [11]. In addition, the study has found that dietary regimens that combine plant proteins and probiotics can specifically enhance muscle health by regulating gut microbiota and related metabolomics characteristics [12]. Therefore, feed formulations can be optimized by combining functional additives such as probiotics or postbiotics to alleviate the adverse reactions on aquatic animals caused by plant protein substitution [13,14].

*Enterococcus faecalis*, as a probiotic of lactic acid bacteria, has been clearly included in the category of “microbial feed additives”. With its acid and bile salt tolerance, strong intestinal colonization ability, and facultative anaerobic metabolic characteristics, it shows unique advantages in shrimp farming compared with traditional probiotics such as *Bacillus subtilis* and *Lactobacillus*. A study has shown that the probiotic *E. faecalis* YFI-G720 significantly elevates health metrics, including immune response markers, intestinal microbiota balance, and overall disease resilience in *Carassius auratus* [15]. The mechanism of action includes competitive inhibition of *Vibrio colonization*, secretion of antimicrobial peptides, and promotion of intestinal villi development [16]. Postbiotics are defined as microbial preparations consisting of inactivated microbial cells and/or their metabolites that confer health benefits to the host [17]. Recently, preparations featuring high safety, stable bioactive composition, and physiological regulatory functions have gained increasing attention as efficient alternatives to live probiotics [18]. They have been reported to exert anti-inflammatory, antioxidant, growth-promoting, and gut health-improving effects [19]. Hasan et al. found that heat-inactivated *Bacillus* sp. SJ-10 had a positive effect on the expression of weight gain rate, protein efficiency, lysozyme, and superoxide dismutase activity in the liver and gill of *Paralichthys olivaceus* [20]. Interestingly, heat-inactivated *Bacillus pumilus* SE5 was more effective in improving the growth performance and immune response of grouper [21]. In previous studies of our group, three mutant strains (EF-448, EF-798, EF-804) with high yield of digestive enzymes and stable genetic properties were obtained by atmospheric and room temperature plasma (ARTP) mutagenesis of *E. faecalis*, and it was confirmed that they had no safety problem to *M. rosenbergii* [22,23]. In this study, these three mutant strains were selected as the candidate strains for the preparation of postbiotics, so as to further screen out better *E. faecalis* for the feed application of *M. rosenbergii*. The effects of dietary supplementation of three different heat-inactivated *E. faecalis* strains on growth performance, antioxidant capacity, intestinal microbiota, and metabolomics characteristics of *M. rosenbergii* in low fish meal diets were evaluated. This study will provide a theoretical basis for further in-depth application of the feed postbiotics.

## 2. Materials and Methods

### 2.1. Postbiotics and Methods

*E. faecalis* 448, 798, and 804 were obtained from the Freshwater Fisheries Research Center of Chinese Academy of Fishery Sciences [22]. The three strains were activated by 1% inoculation in MRS liquid medium at 37 °C, 180 r/min for 24 h. After that, the seed solution of the cultured strain was inoculated at 1% and incubated for 18 h under the same conditions until the end of the logarithmic growth phase. The concentration of the bacterial suspension was adjusted thereafter. Postbiotics were prepared by heat inactivation at 121 °C for 20 min, with subsequent confirmation of the absence of viable bacteria.

The culture experiment was designed as 5 groups: a normal fish meal group as the control group (CT, with a basic fish meal ratio of 30%), and a sesame meal instead of 7% fish meal as the low fish meal group (LF). On the basis of a low fish meal diet, three strains of 1 × 10^8^ CFU g^−1^ heat-inactivated *E. faecalis* (HK-448, HK-798, and HK-804) were added to the three low fish meal groups (LF+HK-448, LF+HK-798, and LF+HK-804).

The composition and nutritional components of the feed used in the experiment are shown in Table 1. All raw materials were comminuted through a 60-mesh sieve and sequentially blended. Subsequently, oil and an appropriate amount of water were added, followed by pelletization of sinking feed using an F-26 twin-screw extruder (Guangzhou Huagong Opto-Mechanical & Electrical Technology Co., Ltd., Guangzhou, China). The pelleting temperature was controlled at 85 ± 5 °C. All feeds were prepared and stored at −20 °C until use.

### 2.2. Prawns and Feeding Trial

*M. rosenbergii* was provided by Zhejiang Nantaihu Freshwater Aquatic Seed Industry Co., Ltd. (Zhejiang, China), and domesticated in Yixing Dapu Base of Freshwater Fishery Research Center of Chinese Academy of Fishery Sciences for two weeks. A total of 450 healthy *M. rosenbergii* (2.85 ± 0.50 g) were randomly distributed into 15 cylindrical tanks (140 cm in diameter; 120 cm in depth) and fed at 5–10% of body weight. These tanks were randomly distributed into five treatments with three replicates per treatment. An appropriate amount of artificial water grass was put in the tank to prevent prawns cannibalism. During the experiment, the water level of the breeding barrel was maintained at 50 ± 5 cm, the water temperature was 26.7 ± 1.5 °C, the pH was 7.4–8.0, dissolved oxygen was 8–9 mg/L, and ammonia nitrogen was <0.05 mg/L. The prawns were fed once at 7:00, 12:00, and 17:00 every day, and the feed intake of prawn was estimated to ensure sufficient feed.

### 2.3. Sample Collection

At the end of the 8-week feeding experiment, the total weight of each barrel of prawns was measured, and the number of prawns was counted to calculate the growth performance. The body length, body weight, hepatopancreas weight, and muscle weight of the prawns were measured to calculate the body index. Hepatopancreas and intestines were collected using sterile instruments. The hepatopancreas from three prawns was pooled as one biological replicate in the group, while intestinal tissues from five prawns constituted one replicate, frozen in liquid nitrogen and stored at −80 °C. The hepatopancreas was used for subsequent antioxidant enzyme determination and transcriptional determination of immune-related genes, part of the intestine was used for intestinal microbiota analysis, and part was used for metabolomics analysis.

### 2.4. Growth Performance

At the beginning and end of the experiment, the prawns were individually weighed and measured to determine their growth performance. The following formulas are used to calculate the growth performance indicators:

Survival rate (SR, %) = (final shrimp number/initial shrimp mber) × 100

Weight gain rate (WGR) = (FBW − IBW)/IBW × 100

Specific growth rate (SGR, %/days) = (In FBW − In IBW)/days × 100

Feed conversion ratio (FCR) = dry feed consumed/weight gain

Condition factor (CF, %) = (W/L^3^) × 100 (W is weight, L is total length)

Hepatosomatic index (HSI, %) = (weight of the livers/body weight) × 100

Flesh percentage (FP, %) = (weight of flesh/total body weight) × 100

### 2.5. Analysis of Antioxidant Capacity

The hepatopancreas samples were homogenized in sterile ice-cold physiological saline, centrifuged (10 min; 3000 rpm; 4 °C), and the resulting supernatant was collected for subsequent enzyme activity assays. The activities of total protein (TP), superoxide dismutase (SOD), total antioxidant capacity (T-AOC), total nitric oxide synthase (TNOS) and inducible nitric oxide synthase (iNOS), and the contents of malondialdehyde (MDA) and nitric oxide (NO) in hepatopancreas were determined by kits from Nanjing Jiancheng Bioengineering Institute (Jiangsu, China, TP kit code: A045-4-2; SOD kit code: A001-3-1, T-AOC kit code: A015-3-1; NOS kit code: A014-1; NO kit code: A013-2-1; MDA kit code: A003-1-1).

### 2.6. RNA Isolation and RT-qPCR Analysis

To determine the expression of related immune genes, total RNA was isolated from hepatopancreas samples using RNAiso Plus Takara Bio Inc., Otsu, Japan and purified with RNase-Free DNase Takara Bio Inc., Otsu, Japan to eliminate genomic DNA contamination. RNA purity and concentration were measured via a NanoDrop device (DN-1000, Thermo Scientific, Waltham, MA, USA). After normalizing RNA concentration, cDNA was synthesized from 500 ng of DNase-treated RNA using the ExScript^TM^ RT-PCR kit Takara Bio Inc., Otsu, Japan following the manufacturer’s protocol.

For RT-qPCR, cDNA samples were analyzed on a BIO-RAD real-time detector (Bio-Rad Laboratories Inc., Hercules, CA, USA) using the SYBR Green II Fluorescence Kit Takara Bio Inc., Otsu, Japan. The reaction mixture (20 μL total) contained 10 μL SYBR^®^ Premix Ex Taq^TM^, 0.4 μL ROX reference dye II, 0.4 μL each of forward and reverse primers (10 μM), 2.0 μL cDNA solution, and 6.8 μL ddH_2_O. Primers (listed in Table 2) were designed using Primer 5 software. The thermal cycling protocol was: initial denaturation at 95 °C for 30 s, followed by 40 cycles of 95 °C for 5 s and 60 °C for 30 s. A melt curve analysis was performed from 95 °C to 60 °C (15 s at 60 °C, then ramped to 95 °C for 15 s) to verify primer specificity. Non-reverse transcribed RNA served as a control to assess background and genomic DNA contamination (which was negligible). β-actin was used as the housekeeping gene, confirmed for stable expression across samples. Each reaction was run in triplicate, and relative gene expression was calculated using the 2^−ΔΔCT^ method [24].

### 2.7. Analysis of Intestinal Microbiota

The intestinal contents of the CT, LF, and LF+ HK-804 groups (5 intestines/replicate, 3 replicates/group) were transported via dry ice to Nanjing Genepioneer Biotechnologies Co., Ltd., Nanjing, China. Total microbial DNA was extracted from the intestinal contents using the E.Z.N.A.^®^ Soil DNA Kit (Omega Bio-Tek, Norcross, GA, USA). Specific primers targeting the V3-V4 region of the 16S rRNA gene were designed to amplify the target region, generating amplicons approximately 420 bp in length. Adapters were ligated, and paired-end sequencing (2 × 250 bp) was performed using the Illumina NovaSeq 6000 platform. For 16S analysis, read quality filtering was conducted via PANDAseq to merge paired-end reads (min overlap: 10 bp; max overlap mismatch ratio: 0.2), followed by PRINSEQ-based trimming of 3′-end bases with Q-score < 20 and discarding of sequences with ≥5% N bases. Sequence statistics were compiled post-filtering. Amplicon sequence variants (ASVs) were resolved from the quality-filtered reads using the DADA2 pipeline in QIIME2. Alpha diversity indices (Chao1, Observed species, and Shannon index) were calculated using QIIME2 software (2021.11). Statistical analyses of microbial community evenness and diversity were performed at the phylum and genus levels. LEfSe (Linear discriminant analysis Effect Size) analysis was performed to identify statistically significantly differentially abundant bacterial taxa from the phylum to genus level between groups, with the linear discriminant analysis (LDA) score threshold set to 4.0 and *p*-value threshold set to 0.05.

### 2.8. Intestinal Metabolome Analysis

In the CT, LF, and LF+HK-804 experimental groups, the intestinal contents of *M. rosenbergii* (50 mg) were carefully extracted using a 400 μL methanol/water solution (4:1, *v*/*v*) to extract metabolites for subsequent determination. The extracted metabolites were analyzed using an advanced Vanquish UHPLC system integrated with an Orbitrap Q Exactive mass spectrometer (all from Thermo Fisher, Waltham, MA, USA), as described by Yu et al. [26]. Proogene QI v3.0.3 was used to process the original data, and peak alignment, deconvolution, and normalization were performed on the QC samples. Missing values were uniformly processed for comparison and QC samples: metabolites with >50% missing rates were excluded, and those with <50% imputed via KNN (R v3.6.2, DMwR package). The MetaboAnalyst R package (3.6.2) was employed to exclude the mass spectrometry features (i.e., metabolites) with a relative standard deviation (RSD) > 30% across all QC samples.

Differential metabolites were identified through integrated univariate and multivariate statistical analyses. Univariate criteria included fold change (FC) ≥ 1.2 or ≤0.8333 and *p*-value < 0.05 (Student’s *t* test). Multivariate analysis employed partial least squares-discriminant analysis (PLS-DA) with variable importance in projection (VIP) scores ≥ 1.0. Putative metabolites were annotated against the Kyoto Encyclopedia of Genes and Genomes (KEGG) PATHWAY database, Human Metabolome Database (HMDB), and LipidMaps using mass tolerance ≤ 5 ppm and retention time matching. Structural validation was performed using MS/MS fragmentation patterns, isotopic similarity scores, and pathway enrichment analysis via KEGG Orthology (KO) mapping.

### 2.9. Statistical Analysis

Except for intestinal microbiome and metabolome data, all statistical analyses were performed using SPSS Statistics 27.0 for One-Way ANOVA and Duncan test for significant difference analysis. Kruskal–Wallis test was specifically applied for intergroup comparisons to evaluate differences in α diversity, intestinal microbiota relative abundance, and intestinal metabolite abundance across our experimental groups, with false discovery rate (FDR) correction implemented to adjust for multiple comparisons. Spearman correlation analysis was performed between the relative abundances of differential bacterial genera and the relative contents of differential metabolites, while Pearson correlation analysis was used to assess the relationships between the relative contents of differential metabolites and the quantitative values of immune-antioxidant parameters. Data are presented as the mean ± standard error of the mean (SEM), and *p* < 0.05 was considered statistically significant.

## 3. Results

### 3.1. Growth Performance and Physical Indicators

As shown in Table 3, compared with the CT group, the WGR and SGR of the LF group were significantly reduced (*p* < 0.05), while the FCR was significantly increased (*p* < 0.05). Compared with those of the LF group, WGR, SGR, FP, and HSI in the group of LF+HK-804 were significantly increased (*p* < 0.05). There was no significant difference in growth performance among the CT, the LF+HK-448 group, and the LF+HK-798 group (*p* > 0.05).

### 3.2. Antioxidant Capacity

The results of the antioxidant capacity of hepatopancreas among different groups are shown in Table 4. Compared with that of the CT group, the content of NO and MDA was significantly increased, and the activities of TNOS and iNOS were significantly decreased in the LF group (*p* < 0.05). The MDA content in the three postbiotic groups was significantly lower than that in the LF group (*p* < 0.05), with no significant difference compared to the CT group (*p* > 0.05); the NO content in the LF+HK-798 and LF+HK-804 groups was significantly higher than that in the LF group (*p* < 0.05); the T-AOC and SOD contents in the group of LF+HK-798 were significantly lower than those in the LF group (*p* < 0.05).

### 3.3. Analysis of Immune-Related Genes

**Figure 1:**
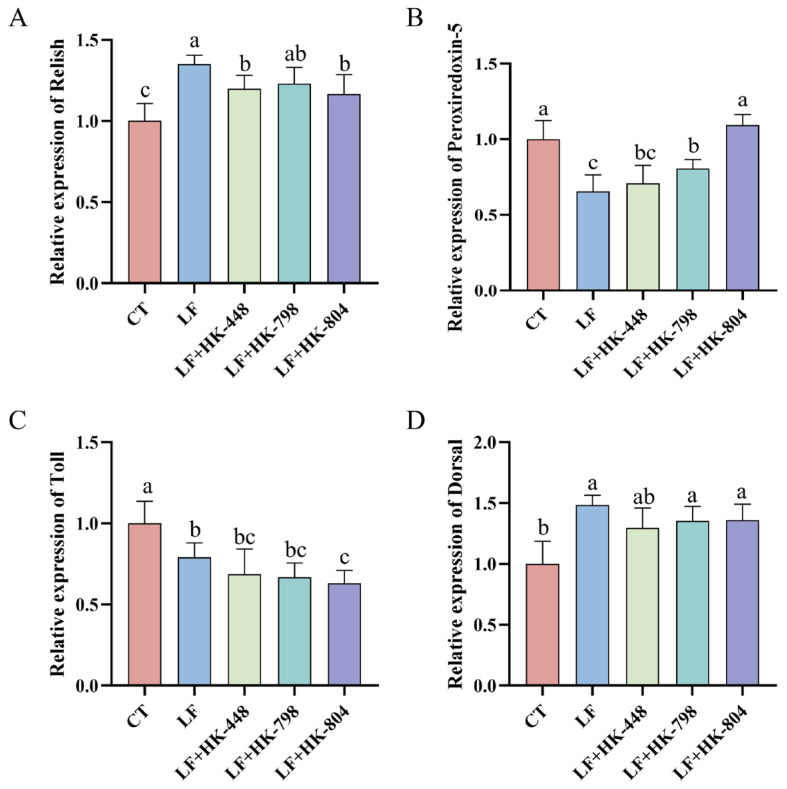
The effects of three heat-inactivated *E. faecalis* strains in the low fish meal feed on the relative expression of Relish (**A**), Peroxiredoxin-5 (**B**), Toll (**C**), and Dorsal (**D**) in the intestine of *M. rosenbergii*. The value is mean ± SEM (n = 8). Different letters indicate significant differences (*p* < 0.05).

As shown in Figure 1, compared with that of the CT group, the relative expressions of *Relish* and *Dorsal* genes in the LF group were significantly up-regulated (*p* < 0.05), while the relative expressions of *Peroxiredoxin-5* and *Toll* genes were significantly down-regulated (*p* < 0.05). After supplementation with heat-inactivated *E. faecalis*, compared with that of the LF group, the relative expression of *Relish* in the group of LF+HK-448 and LF+HK-804 was significantly down-regulated (*p* < 0.05); the relative expression of *Peroxiredoxin-5* gene in the group of LF+HK-798 and LF+HK-804 was significantly up-regulated (*p* < 0.05); the relative expressions of *Toll* genes in the LF+HK-804 group were significantly down-regulated (*p* < 0.05).

### 3.4. Intestinal Microbial Diversity

**Figure 2:**
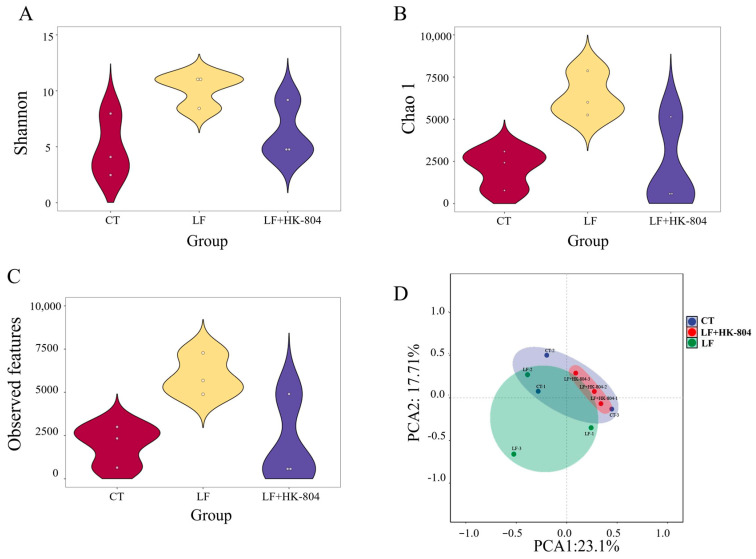
Effects of heat-inactivated *E. faecalis* strains-804 in the low fish meal feed on the alpha and beta diversity of intestinal microbiota in *M. rosenbergii*. alpha diversity was evaluated by the index of (**A**) Shannon, (**B**) Chao1, (**C**) Observed features, and (**D**) Beta diversity was evaluated by principal component analysis (PCA).

Intestinal microbial diversity is shown in Figure 2. In terms of α-diversity, the Shannon, Chao1, and Observed features indices in the LF group were higher than those in the CT group. Compared with those of the LF group, the three α-diversity indices in the LF+HK-804 group were reduced (Figure 2A–C). According to the PCA diagram of β-diversity (Figure 2D), it was noted that the intestinal microbial communities had partial overlaps but relative aggregation areas among the CT group, the LF group, and the LF+HK-804 group, and the intestinal microbial community of the LF+HK-804 group showed a nested distribution within the CT group.

### 3.5. Intestinal Microbial Community Structure Composition

**Figure 3:**
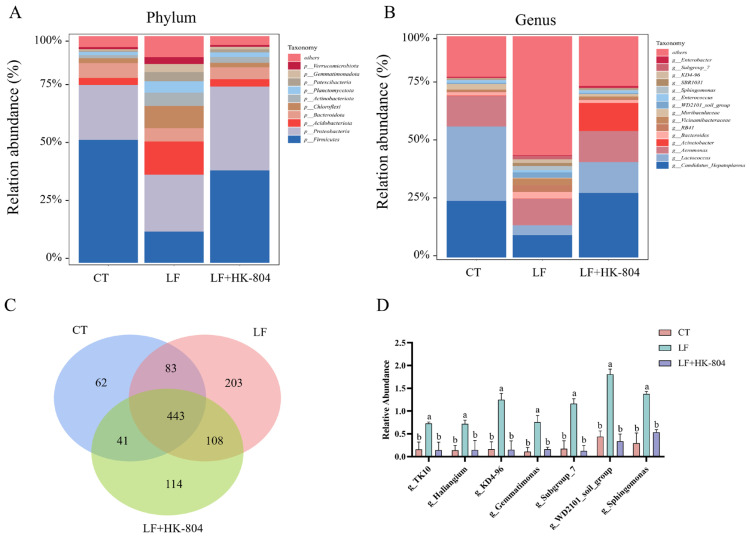
Effects of heat-inactivated *E. faecalis* strains-804 in the low fish meal feed on intestinal microbial composition of *M. rosenbergii*. (**A**) The composition of the top 10 intestinal microbiota at the level of the most abundant phylum of *M. rosenbergii*. (**B**) The composition of intestinal microbiota in *M. rosenbergii* was among the top 15 most abundant genus-level. (**C**) Compare the Venn diagram of intestinal microbiota distribution at the genus level. (**D**) Column diagram of candidate differential bacteria in three groups of common genera. Different lowercase letters indicate significant differences among groups (*p* < 0.05).

The intestinal microbiota of *M. rosenbergii* at the phylum and genus levels are shown in Figure 3. Firmicutes, Proteobacteria, Acidobacteria, Bacteroidetes, and Chloroflexi were the dominant phyla in all samples (Figure 3A). Further analysis at the phylum level showed that compared with those in the CT group, the relative abundances of Acidobacteriota, Chloroflexi, and Verrucomicrobiota in the LF group were significantly increased; whereas compared with those in the LF group, the relative abundances of these three phyla in the LF+HK-804 group significantly decreased.

At the genus level, *Hepatoplasma*, *Lactococcus*, *Aeromonas*, *Acinetobacter*, *Enterococcus,* and *Bacteroides* were dominant in all samples. Compared with the other groups, the LF group exhibited a significantly lower relative abundance of *Candidatus Hepatoplasma*, *Lactococcus*, and *Enterococcus*, while showing a significantly higher relative abundance of *WD2101_soil_group*, *Sphingomonas*, *KD4-96*, and *Subgroup_7* (Figure 3B).

According to the Venn diagram (Figure 3C), 443 common genera were observed in the three groups, while 526 genera were observed in the CT group and the LF group; there were 551 genera in the LF group and the LF+HK-804 group. In the three groups of common genera, CT vs. LF and LF vs. LF+HK-804 were further subjected to an independent sample T test.

Seven genera were screened out in the two groups, and the relative abundance was greater than 0.1%. The columnar diagram showed that *TK10*, *Haliangium*, *KD4-96*, *Gemmatimonas*, *Subgroup_7*, *WD2101_soil_group*, and *Sphingomonas* all showed a significant increase in the relative abundance in the LF group compared to those of the CT group (*p* < 0.05). The significant decrease in the relative abundance in the LF+HK-804 group compared to that of the LF group (*p* < 0.05, Figure 3D).

### 3.6. Differences in Intestinal Microbial Composition

**Figure 4:**
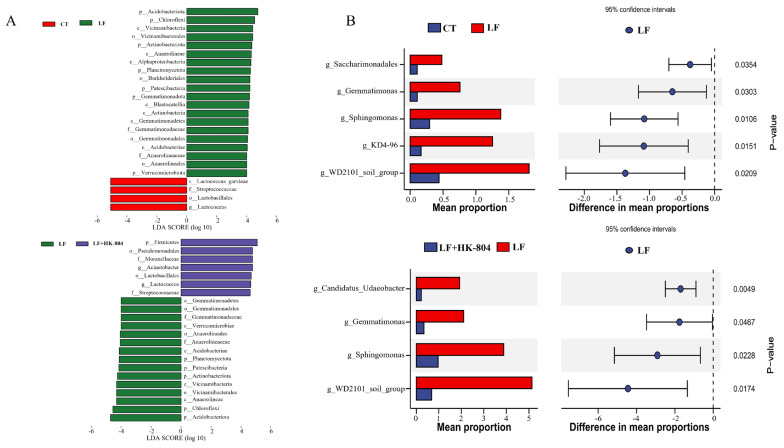
Analysis of microbial differences between groups. (**A**) LEfSe analysis of CT and LF, LF and LF+HK-804. The histogram represents the LDA score after logarithmic transformation between groups, and the threshold is LDA > 4.0. (**B**) Bar charts of horizontal microbial changes between CT and LF, LF and LF+HK-804.

The results of LEfSe analysis showed that the microbiota was significantly altered (Figure 4A). When comparing the microbiota between the CT and LF groups, four taxonomic classifications were markedly changed in the CT group, while 20 classifications were altered in the LF group. In the comparison between the LF and LF+HK-804 groups, 15 and seven significantly changed classifications were observed in the LF and LF+HK-804 groups, respectively. Differentially abundant taxa showing changes in the LF group compared with the CT and LF+HK-804 groups included five phyla (Planctomycetota, Patescibacteria, Actinobacteriota, Chloroflexi, and Acidobacteriota), four classes (Gemmatimonadetes, Acidobacteriae, Vicinamibacteria, and Anaerolineae), three orders (Gemmatimonadales, Anaerolineales, and Vicinamibacterales), and two families (Gemmatimonadaceae and Anaerolineaceae).

Subsequently, STAMP analysis was performed to identify and visualize genera with significant differences (Figure 4B). Compared with that of the CT group, the abundances of *Saccharimonadales*, *Gemmatimonas*, *Sphingomonas*, *KD4-96*, and *WD2101_soil_group* were significantly increased in the LF group (*p* < 0.05). Compared with that of the LF group, the abundances of *Candidatus_Udaeobacter*, *Gemmatimonas*, *Sphingomonas*, and *WD2101_soil_group* significantly decreased in the LF+HK-804 group (*p* < 0.05).

### 3.7. Intestinal Metabolome Analysis

**Figure 5:**
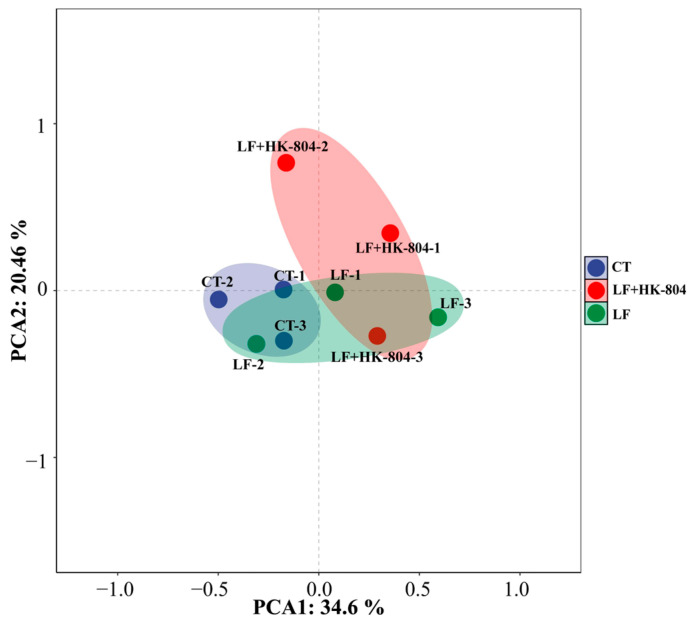
PCA of intestinal metabolite.

PCA analysis was performed to evaluate the differences in metabolites among the CT, LF, and LF+HK-804 groups (Figure 5). In terms of the trend of inter-group separation, the distributions of the CT group and the LF group have partial overlap but are clearly distinguishable; the LF group and the LF+HK-804 group have a small amount of overlap but are significantly separated as a whole.

**Figure 6:**
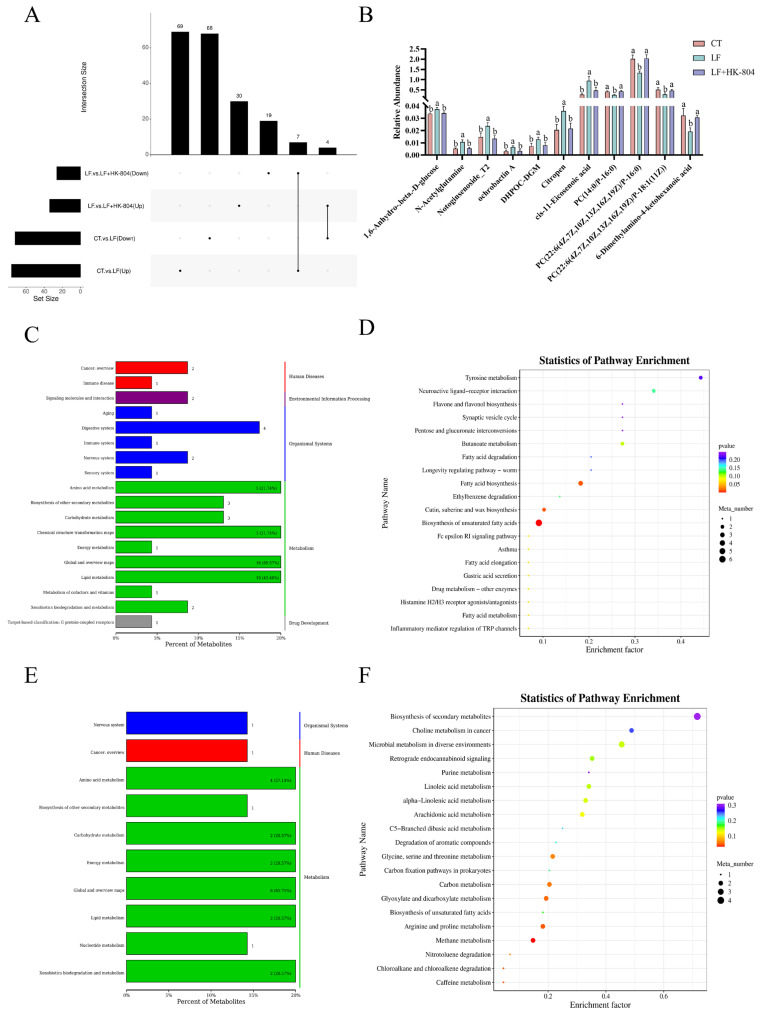
The metabolites and KEGG pathway-related analysis results were compared between CT vs. LF, LF vs. LF+HK-804. (**A**) Upset diagram of differential metabolites; (**B**) Columnar diagram of differential metabolites; (**C**) KEGG pathway classification of CT vs. LF (left: secondary classification; right: first-level classification); (**D**) KEGG enrichment of CT vs. LF; (**E**) KEGG pathway classification of LF vs. LF+HK-804 (left: secondary classification; right: first-level classification); (**F**) KEGG enrichment of LF vs. LF+HK-804. Different lowercase letters indicate significant differences among groups (*p* < 0.05).

Figure 6 combines the results of the upset Wayne diagram, differential metabolite histogram, Percent of Metabolites and Statistics of Pathway Enrichment. With VIP > 1 and *p* < 0.05 as the criteria for screening differential metabolites, 148 annotated differential metabolites (76 up-regulated and 72 down-regulated) were detected in the CT group and LF group. A total of 60 annotated differential metabolites (34 up-regulated and 26 down-regulated) were detected in the LF group and LF+HK-804 group (Figure 6A). Based on the common differential metabolites in the CT vs. LF and LF vs. LF+HK-804 groups as screening conditions, a total of 11 differential metabolites were screened. Compared with the LF group, there were 1,6-Anhydro-.beta.-D-glucose, N-Acetylglutamine, Notoginsenoside_T2, ochrobactinA, 5.7-Dihydroxy-2-(4-hydroxyphenyl)-4-oxo-4H-chro-men-3-yl6-deoxy-2-O-.beta.-D-glucopyranosyl-.alpha.-L-mannopyranoside (DHPOC-DGM), Citropen, and cis-11-Eicosenoic acid were significantly down-regulated in the CT group and LF+HK-804 group (*p* < 0.05). Four metabolites of PC (14:0/P-16:0), PC (22:6(4Z, 7Z, 10Z, 13Z, 16Z, 19Z)/P-16:0), PC (22:6 (4Z, 7Z, 10Z, 13Z, 16Z, 19Z)/P-18:1(11Z)), and 6-Dimethylamino-4-ketohexanoic acid were significantly up-regulated in CT group and LF+HK-804 group (*p* < 0.05; Figure 6B).

In the KEGG pathway type classification diagram, a total of 61 differential metabolites in CT vs. LF were annotated to the KEGG pathway (Figure 6C,E). These metabolites were annotated into five categories: metabolism (46), body system (9), human disease (3), environmental information processing (2), and drug development (1). A total of 22 differential metabolites in LF vs. LF+HK-804 were annotated to the KEGG pathway, and these metabolites were annotated into three categories: metabolism (20), body system (1), and human disease (1).

The results of KEGG pathway enrichment analysis showed that the differential metabolites in the CT vs. LF group were significantly enriched in Fatty acid biosynthesis, Cutin, Suberine, and Wax biosynthesis pathways (*p* < 0.05), and extremely significantly enriched in Biosynthesis of unsaturated fatty acid (*p* < 0.001). The differential metabolites in the LF vs. LF+HK-804 group were significantly enriched in Methane metabolism, Caffeine metabolism, Chloroalkane and chloroalkene degradation, Arginine, and proline metabolism pathways (*p* < 0.05; Figure 6D,F).

### 3.8. Correlation Analysis

**Figure 7:**
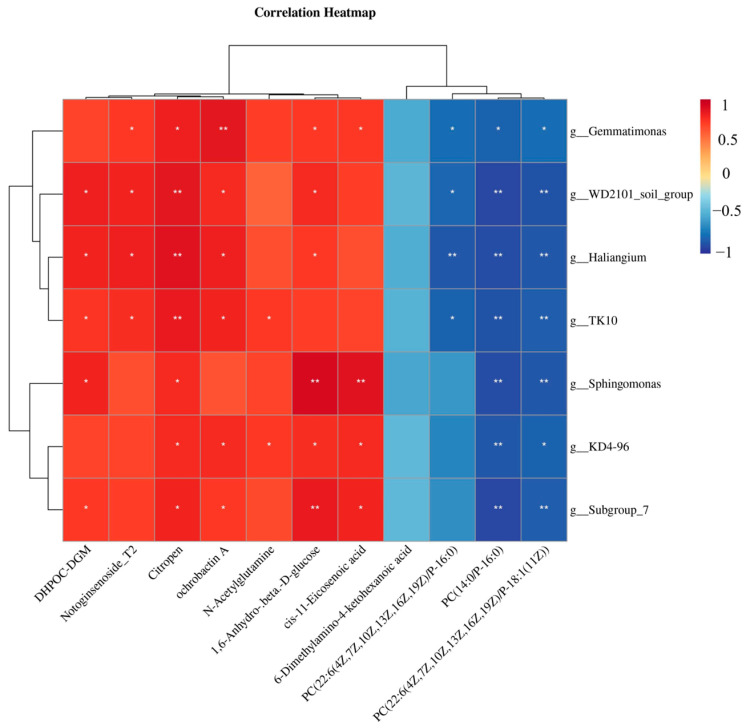
Heat map of the correlation between intestinal microbiota and differential metabolites. The abscissa represents intestinal metabolites, and the ordinate represents intestinal microorganisms. Each box in the figure represents the correlation between the two factors. Red represents positive correlation, blue represents negative correlation. Asterisks indicate significant differences: * *p* < 0.05, ** *p* < 0.01.

The results of association analysis between 11 intestinal differential metabolites shared by the CT vs. LF group, LF vs. LF+HK-804 group, and seven intestinal differential microbial genera are shown in Figure 7. *Gemmatimonas* was significantly positively correlated with Notoginsenoside_T2, Citropen, ochrobactin A, 1,6-Anhydro-.beta.-D-glucose and cis-11-Eicosenoic acid (*p* < 0.05). There was a significant negative correlation with organic acid phospholipids (*p* < 0.05). *WD2101_soil_group* was significantly positively correlated with DHPOC-DGM, Notoginsenoside_T2, Citropen, ochrobactin A and 1,6-Anhydro-.beta.-D-glucose (*p* < 0.05), and significantly negatively correlated with organic acid phospholipids (*p* < 0.05). *Sphingomonas* was positively correlated with DHPOC-DGM, Citropen, 1,6-Anhydro-.beta.-D-glucose and cis-11-Eicosenoic acid (*p* < 0.05), and negatively correlated with PC (22:6 (4Z, 7Z, 10Z, 13Z, 16Z, 19Z)/P-16:0), PC (22:6 (4Z, 7Z, 10Z, 13Z, 16Z, 19Z)/P-18:1 (11Z)) (*p* < 0.05).

**Figure 8:**
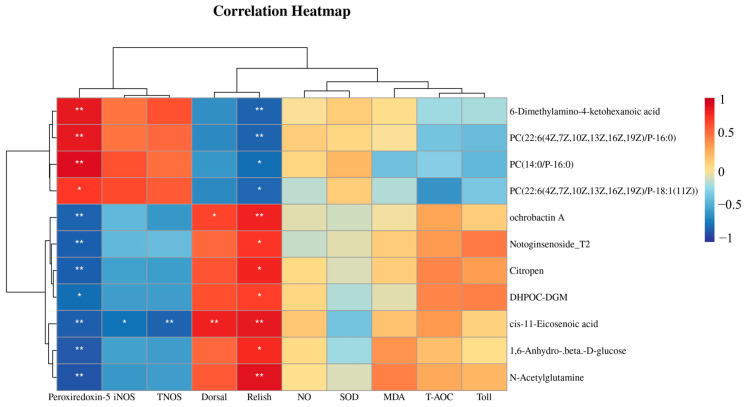
Heat map of the correlation between intestinal differential metabolites and gene indicators, and the ordinate represents the intestinal differential metabolites. Each box in the figure represents the correlation between the two. Red represents a positive correlation, and blue represents a negative correlation. Asterisks indicate significant differences: * *p *< 0.05, ** *p* < 0.01.

As shown in Figure 8, the correlation between immune genes, antioxidant indicators, and intestinal differential metabolites was analyzed. The results showed that 6-dimethylamino-4-ketohexanoic acid, PC (22:6 (4Z, 7Z, 10Z, 13Z, 16Z, 19Z)/P-16:0), PC (22:6 (4Z, 7Z, 10Z, 13Z, 16Z, 19Z)/P-18:1 (11Z)), and PC (14:0/P-16:0) were positively correlated with the expression of *Peroxiredoxin-5*, iNOS, and TNOS, but negatively correlated with the expression of *Dorsal* and *Relish*. In contrast, ochrobactin A, notoginsenoside T2, citropen, DHPOC-DGM, cis-11-eicosenoic acid, 1,6-anhydro-β-D-glucose, and N-acetylglutamine were negatively correlated with the expression of *Peroxiredoxin-5*, iNOS, and TNOS, while positively correlated with the expression of *Dorsal* and *Relish*.

## 4. Discussion

At present, global fish meal resources are facing the dual pressures of fishing quota restrictions [27]. The aquaculture industry’s rigid dependence on fish meal has forced the industry to urgently develop sustainable protein alternatives. Various plant proteins are used to partially or completely replace fish meal in aquatic feeds due to their cost-effectiveness and sustainability [28,29]. However, if the proportion of plant protein substitution is not properly regulated, it will easily lead to the imbalance of amino acid composition and the accumulation of anti-nutritional factors, which will adversely affect the growth performance of aquatic animals [30]. The introduction of postbiotics as a functional feed additive into the plant protein substitution system can more effectively alleviate the negative effects, such as growth inhibition caused by plant protein substitution, than traditional probiotics [5,31].

In the low fish meal model replaced by sesame meal constructed in this study, the anti-nutritional factors such as phytic acid and oxalic acid contained in sesame meal inhibited the growth of *M. rosenbergii* to a certain extent, and the feed coefficient decreased. Among these three heat-inactivated *E. faecalis* strains, *E. faecalis* 804 showed the better growth compensation effect, which significantly alleviated the down-regulation of weight gain rate and specific growth rate, and increased meat content and hepatosomatic ratio. Similar to the study, heat-inactivated *Bacillus pumilus* SE5 significantly increased the weight gain and SGR of grouper *Epinephelus coioides*, and significantly reduced FCR [20]. The addition of *E. faecalis* Y17 in the mud crab (*Scylla paramamosain*) can improve the growth performance [32]. However, there was no significant difference in survival rate and condition factor among the three groups, which may be related to the stress intensity of fish meal replacement or the corresponding physiological threshold of the experimental period [33]. The different alleviating effects of three heat-inactivated *E. faecalis* strains on the growth inhibition of *M. rosenbergii* may be consistent with the research logic of metabolic differences in ARTP-mutagenized strains. Specifically, ARTP mutagenesis may alter the expression of metabolic genes of microorganisms, leading to changes in metabolites. The inherent differences in their metabolic pathways are still reflected in the form of ‘differences in residual bioactive substances’ after heat inactivation, which ultimately results in distinct functional effects [34]. The secretion efficiency of recombinant protein in the ARTP mutant of *Bacillus subtilis* was significantly increased, which was attributed to the enhanced extracellular protein accumulation ability [35]. The ARTP and ALE strategies were successfully used to achieve stable and heritable changes in the metabolic pathways of *Escherichia coli* strains, which were directly manifested as the enhancement of anaerobic succinic acid biosynthesis [36]. These results indicate that heat-inactivated *E. faecalis* 804 has a growth-promoting effect and has the potential to be used as a functional feed additive in aquatic feed.

Antioxidant system and immune regulation play a key role in determining the health status of crustaceans [37]. The content of MDA in the LF group increased, indicating that a low fish meal diet may aggravate lipid peroxidation damage, and the body may compensate through the non-enzymatic antioxidant system to maintain the overall antioxidant capacity. However, feeding three postbiotics groups can effectively alleviate lipid peroxidation damage in this study. It has been reported that supplementation of 500 mg/kg *E. faecium* feed can reduce MDA and enhance antioxidant capacity [38]. Similar results were observed in *M. rosenbergii* fed with different doses of *Clostridium butyricum* [39,40].

Unlike typical antioxidant enzymes, NOS catalyzes the NO product in organisms, which has the function of resisting disease and controlling immunity [41]. The content of NO in the LF group was significantly higher than that in the CT group, but the activities of TNOS and iNOS were significantly decreased, suggesting that a low fish meal diet may break the balance of NO metabolism in aquatic animals through the non-enzymatic pathway, and the negative feedback regulation of iNOS cannot offset the increase in NO in the non-enzymatic pathway. However, the LF+HK-798 and LF+HK-804 groups significantly increased NO content, which may restore balance by relieving iNOS negative feedback inhibition and regulating NO metabolic pathway, while other postbiotics groups did not change NO content, indicating that the regulation of postbiotics on NO metabolism is strain-specific, and its mechanism needs to be further explored. These results indicate that the addition of heat-inactivated *E. faecalis* 804 can reduce the oxidative damage of hepatopancreas in *M. rosenbergii*.

Low fish meal diets have been shown to induce stress responses in *M. rosenbergii*, which may lead to inflammation [42]. In order to further explore the mechanism of heat-inactivated *E. faecalis* in alleviating inflammatory response, this study also explored the relative expression of *Relish*, *Peroxiredoxin-5*, *Toll*, and *Dorsal* genes. The results showed that compared with the LF group, the relative expression levels of *Relish* and *Toll* genes in prawn fed with heat-inactivated *E. faecalis* diets were relatively low. In addition, the relative expression level of *Peroxiredoxin-5* in the LF+HK-804 group was significantly higher than that in the LF groups. The results showed that all three heat-inactivated *E. faecalis* strains could alleviate the adverse effects of low fish meal diets to varying degrees, among which HK-798 and HK-804 had better regulatory effects, especially the HK-804 strain, which could improve immune disorders and antioxidant deficiencies.

A previous study has found that *Dorsal* and *Relish* genes of *M. rosenbergii* are the core functional genes in the innate immune system, and have high sequence similarity with mammalian homologous genes [25]. Both of them participate in the signal transduction process of the innate immune pathway; as the upstream regulatory factor of the immune pathway, the expression level of the *Toll* gene can directly regulate the activation state of the downstream *Dorsal* and *Relish* genes [43]. At the same time, *peroxiredoxin 5* belongs to the peroxidase family, which not only enhances the body’s tolerance to oxidative stress but also plays an important regulatory role in the immune response [44]. *E. faecalis* can modulate host immune gene expression, particularly in pathways related to antimicrobial peptide production and inflammation, thereby helping to fine-tune the balance between immune activation and tolerance [45]. Combined with the results of TNOS and NO content, it is very likely that dietary supplementation of *E. faecalis* may activate the Toll–Dorsal pathway and induce the production of NO, thereby eliminating oxidative free radicals and enhancing its antioxidant capacity and immunity.

Intestinal microbiota have a crucial impact on the maintenance of normal physiological functions of the intestine and the resistance to external harmful factors [46,47,48]. A stable and favorable microbial composition is beneficial to the growth performance and health status of the host [49]. In this study, it was found that heat-inactivated *E. faecalis* 804 could reverse the dysbacteriosis induced by low fish meal. The low fish meal diet may destroy the colonization resistance of the microbiota and lead to the excessive proliferation of non-dominant bacteria, while the addition of *E. faecalis* can alleviate the dysbacteriosis. The abundance of Firmicutes in the LF group was significantly reduced, and the LF+HK-804 group could partially restore the level of Firmicutes. Firmicutes bacteria provided a good index of intestinal status, and related studies have confirmed that *Lactobacillus* and *Lactococcus* in Firmicutes can effectively improve the immune gene expression and disease resistance of *Oreochromis niloticus* and *Oncorhynchus mykiss* [50]. Differential analysis showed that *E. faecalis* could specifically regulate the abundance of bacteria such as *Gemmatimonas*, *WD2101_soil_group*, and *Sphingomonas*. It may improve the health of the prawn by participating in the host’s antioxidant pathway [51] or intestinal barrier regulation [52]. According to previous studies, *Gemmatimonas* can dissolve insoluble elements, induce plant resistance, or produce antifungal antibiotics [53]. Members of the phylum Planctomycetota (e.g., *WD2101_soil_group*) possess diverse hydrolytic capabilities and high glycolytic potential, and may be involved in the degradation of plant-derived polymers, exoskeletons of peat-dwelling arthropods, and extracellular polysaccharides produced by other bacteria [54]. In addition, *Sphingomonas* strains can be used as probiotics, which significantly improve the survival rate and growth performance of *Labeo rohita* fingerlings challenged by *Vibrio anguillarum*, and enhance their key biochemical components in vivo, thus having the application potential to prevent fish vibriosis [55]. These results indicate *E. faecalis* may alleviate the negative impact of a low fish meal diet on the intestinal microecology of *M. rosenbergii* by restoring the diversity of microbiota, reshaping the structure of dominant microbiota, and regulating the abundance of functional microbiota.

Metabolomics is a subject that studies the composition, content, and dynamic changes in endogenous small molecule metabolites under the influence of environmental stimulation, pathophysiological changes, or genetic variation [56]. This study showed that the 11 key differential metabolites screened out are involved in pathways such as amino acid metabolism and energy metabolism. Ochrobactin A and citric acid affect the composition of intestinal microorganisms, promote the growth of probiotics, and inhibit pathogens, which is consistent with the study of the Chinese mitten crab [57,58]. Notoginsenoside_T2 and flavonoid glycosides can enhance the activity of SOD and GSH-Px, and prevent ammonia and oxidative stress in prawn [59]. N-acetylglutamine supports intestinal epithelial health and immune function [60,61]. Studies have also found that cis-11-eicosenoic acid and PC optimized lipid utilization in a low fish meal diet and improved the growth performance of *Penaeus vannamei* [60,61,62]. In addition, TCA cycle intermediates (such as citrate) are the core of energy production under environmental pressure [63]. Heat-inactivated *E. faecalis* may improve the physiological disorders induced by low-fish meal diets (such as optimization of intestinal health, antioxidant capacity, lipid metabolism, and energy supply) by regulating the metabolism of the aforementioned metabolites, thus providing a metabolic basis for the application of postbiotics [64].

Our study specifically investigated the interaction between specific gut microbiota and gut differential metabolites, and found that *Gemmatimonas*, *WD2101_soil_group*, and *Sphingomonas* are the core genera that regulate the metabolic balance of the prawn gut. Functional convergence and are negatively correlated with phospholipid metabolites and positively correlated with glycoside/fatty acid metabolites. This is consistent with previous studies, which found that *B.coagulans* treatment induced a callback in Firmicutes abundance [65]. Correlation analysis established a significant positive correlation between differential genera (*Sphingomonas*, *Bacillus*, and *Ralstonia*) and secondary metabolites (including sphingosine, dehydrophytosphingosine, amino acid metabolites, etc.) [42]. Studies have also shown that dietary supplementation of *Bacillus* is beneficial to host growth, intestinal microbial homeostasis, anti-stress, and immune and anti-inflammatory responses [66,67]. In this study, the metabolic association between *Gemmatimonas* and *WD2101_soil_group* was highly consistent, both of which were involved in metabolic regulation by promoting the synthesis/accumulation of glycosides and fatty acid metabolites and inhibiting the production of phospholipids. Although *Sphingomonas* has some differences in related metabolites, the core association trend is consistent with the first two genera and reflects the uniqueness of flavonoid glycoside metabolism regulation. These three genera can participate in the physiological response of prawn to changes in feed conditions by regulating the balance of intestinal glycosides, fatty acids, and phospholipid metabolites, or are the key functional genera to maintain the intestinal metabolic homeostasis of prawn under a low fish meal diet.

This study revealed significant associations between differential intestinal metabolites and immune as well as antioxidant parameters. Lysophosphatidylcholines (LysoPCs) containing polyunsaturated fatty acids and 6-dimethylamino-4-ketohexanoic acid were positively correlated with the expression of iNOS, TNOS, and *Peroxiredoxin-5*, suggesting their likely role in triggering an immune–antioxidant synergistic response. As known bioactive lipid signaling molecules, LysoPCs may promote the burst synthesis of NO through yet unidentified pathways [68]. One study demonstrated that dietary supplementation of phospholipids significantly enhanced the immune response and disease resistance of *Procambarus clarkii*, which was associated with modulated immune parameters [69]. The resulting oxidative stress may subsequently upregulate the expression of *Peroxiredoxin-5* and other antioxidants to maintain cellular redox homeostasis. In *Eriocheir sinensis*, LPS-induced NO burst was shown to upregulate *Peroxiredoxin-5* via the MAPK/Nrf2 pathway, underscoring the compensatory role of antioxidant enzymes in response to NO-mediated oxidative stress [70].

In contrast, metabolites such as ochrobactin A, notoginsenoside T2, and N-acetylglutamine exhibited an inverse correlation pattern, indicating the potential presence of distinct immune regulatory strategies in *M. rosenbergii*. *Dorsal* and *Relish*, as core transcription factors of the Toll and IMD pathways, typically govern the expression of antimicrobial peptides (AMPs) [71]. Our findings suggest that when the immune response is predominantly driven by the Dorsal/Relish axis, the iNOS/NO-mediated immune module may be suppressed. This interpretation is supported by observations in *Macrobrachium nipponense*: upon Aeromonas sobria infection, activation of the Dorsal pathway up-regulated AMP expression while concurrently suppressing iNOS activity; conversely, during fungal infection with *Metarhizium anisopliae*, the iNOS/NO pathway dominated, and Dorsal/Relish activity was reduced [72]. Notably, plant-derived metabolites like notoginsenoside T2 and microbial-associated metabolites such as ochrobactin A showed prominent correlation with the two immune-oxidative axes, implying their potential as endogenous modulators that fine-tune the balance between AMP synthesis and NO-mediated defense in *M. rosenbergii* [73]. This differential correlation pattern provides novel insights into how intestinal metabolites coordinate the host’s innate immune resources, potentially enabling *M. rosenbergii* to adapt to diverse microbial stimuli by prioritizing appropriate defense responses.

## 5. Conclusions

This study showed that heat-inactivated *E. faecalis* 804 after ARTP mutagenesis had probiotic properties in *M. rosenbergii* culture. The addition of postbiotics can effectively promote the growth of the prawns, reduce the oxidative damage induced by plant protein, enhance immunity, increase the abundance of intestinal beneficial microbiota, reshape metabolites profile, and maintain intestinal health. In summary, heat-inactivated *E. faecalis* 804 has the potential to be used as a feed additive to promote growth in prawn farming.

## Figures and Tables

**Table 1:** Ingredients and proximate chemical composition of the experimental diets.

Ingredients	CT	LF	LF+HK-448	LF+HK-798	LF+HK-804
Fish meal ^a^	30.00	23.00	23.00	23.00	23.00
Chicken powder	5.00	5.00	5.00	5.00	5.00
Cuttlefish paste	3.00	3.00	3.00	3.00	3.00
Shrimp powder	6.00	6.00	6.00	6.00	6.00
Soybean meal	19.00	19.00	19.00	19.00	19.00
Sesame meal ^b^	0.00	10.00	10.00	10.00	10.00
Rapeseed meal	10.00	10.00	10.00	10.00	10.00
Alpha starch	20.00	16.30	16.30	16.30	16.30
Soybean oil	1.00	1.70	1.70	1.70	1.70
Phospholipid oil	1.00	1.00	1.00	1.00	1.00
Choline chloride (50%)	1.00	1.00	1.00	1.00	1.00
Vitamin and mineral premix ^c^	1.00	1.00	1.00	1.00	1.00
Zeolite powder	1.00	1.00	1.00	1.00	1.00
Calcium dihydrogen phosphate	2.00	2.00	2.00	2.00	2.00
Postbiotics (1 × 10^8^ CFU/g)			HK-448	HK-798	HK-804
Total	100	100	100	100	100
Proximate composition analysis (%)					
Crude protein	40.27	40.25	40.23	40.22	40.24
Ether extract	7.02	7.21	7.20	7.23	7.22
Nitrogen free extract	30.66	29.54	29.54	29.55	29.53
Crude fiber	3.77	5.16	5.15	5.16	5.14
Ash	12.39	11.23	11.21	11.22	11.22
Lys	2.54	2.21	2.21	2.22	2.22
Met-Cys	1.24	1.08	1.10	1.08	1.09
Arg	1.86	1.61	1.63	1.61	1.62

^a^ Crude protein (CP) content of fish meal: 65.57%. ^b^ CP content of sesame meal: 46%. ^c^ Multivitamin and multimineral (IU, g or mg kg ^−1^ of diet: Vitamin A, 25,000 IU; Vitamin D3, 20,000 IU; Vitamin E, 0.2 g; pyridoxine hydrochloride, 0.04 g; vitamin B12, 0.2 mg; inositol, 1 g; vitamin C, 2 g; choline, 2 g; dicalcium phosphate, 20 g; sodium chloride, 2.6 g; magnesium sulfate, 30 mg; sodium selenate, 20 mg; cobalt chloride, 50 mg; potassium iodide, 4 mg).

**Table 2:** Primer sequences for real-time quantitative PCR.

Gene	Primer Sequences (50–30)	Length (bp)	Amplification Length (bp)	Sequence Source
Relish	GATGAGCCTTCAGTGCCAGA	20	238	KR827675.1
	CCAGGTGACGCCATGTATCA	20		
Peroxiredoxin-5 *	ACTGTGTCACCTTGCCATCTT	21	94	Transcriptome data
	AAATCCCTTGGGCTGGAACAA	21		
Toll	TTCGTGACTTGTCGGCTCTC	20	227	KX610955.1
	GCAGTTGTTGAAGGCATCGG	20		
Dorsal	TCAGTAGCGACACCATGCAG	20	200	KX219631.1
	CGAGCCTTCGAGGAACACTT	20		
β-actin	TCCGTAAGGACCTGTATGCC	20	96	AY651918.2
	TCGGGAGGTGCGATGATTTT	20		

* The sequence of *Peroxiredoxin-5* was obtained from the transcriptome data of *M. rosenbergii* in our group [25].

**Table 3:** Growth performance and morphological indices of *M. rosenbergii* fed with three heat-inactivated *E. faecalis* strains for 8 weeks.

	CT	LF	LF+HK-448	LF+HK-798	LF+HK-804
IBW (g)	2.83 ± 0.10	2.79 ± 0.10	2.91 ± 0.04	2.90 ± 0.02	2.93 ± 0.03
FBW (g)	22.64 ± 0.96	19.49 ± 0.73	20.31 ± 0.09	20.68 ± 0.18	22.83 ± 0.47
SR (%)	72.22 ± 4.84	68.89 ± 6.76	63.33 ± 1.93	62.22 ± 2.22	65.56 ± 2.94
WGR (%)	701.57 ± 21.58 ^a^	599.42 ± 1.20 ^b^	598.02 ± 6.70 ^b^	614.15 ± 9.70 ^b^	679.48 ± 11.18 ^a^
SGR (%/d)	3.93 ± 0.05 ^a^	3.67 ± 0.01 ^b^	3.66 ± 0.02 ^b^	3.71 ± 0.03 ^b^	3.88 ± 0.03 ^a^
FCR (%)	1.25 ± 0.02 ^c^	1.52 ± 0.02 ^ab^	1.66 ± 0.06 ^a^	1.61 ± 0.08 ^ab^	1.42 ± 0.09 ^bc^
CF (%)	1.11 ± 0.01	1.12 ± 0.04	1.14 ± 0.02	1.13 ± 0.01	1.14 ± 0.02
HSI (%)	5.36 ± 0.21 ^b^	5.51 ± 0.17 ^b^	6.15 ± 0.52 ^ab^	6.489 ± 0.32 ^ab^	6.87 ± 0.56 ^a^
FP (%)	28.67 ± 0.50 ^ab^	26.81 ± 0.92 ^b^	28.51 ± 0.80 ^ab^	28.13 ± 0.54 ^ab^	30.21 ± 0.65 ^a^

The data were expressed as means ± SEM (n = 3). Different letters indicate significant differences (*p* < 0.05). IBW: initial body weight; FBW: final body weight; SR: survival rate; WGR: weight gain rate; SGR: specific growth rate; FCR: feed conversion ratio; CF: condition factor; HSI: hepatosomatic index; FP: flesh percentage.

**Table 4:** Antioxidant capacity of the hepatopancreas in *M. rosenbergii* fed three heat-inactivated *E. faecalis* strains for 8 weeks.

	CT	LF	LF+HK-448	LF+HK-798	LF+HK-804
T-AOC (mmol/gprot)	0.09 ± 0.01 ^bc^	0.12 ± 0.01 ^ab^	0.11 ± 0.01 ^ab^	0.08 ± 0.01 ^c^	0.13 ± 0.01 ^a^
TNOS (U/mgprot)	9.38 ± 0.34 ^a^	3.16 ± 0.52 ^b^	2.87 ± 0.39 ^b^	3.81 ± 0.77 ^b^	4.11 ± 0.25 ^b^
iNOS (U/mL)	9.48 ± 0.47 ^a^	5.95 ± 0.55 ^bc^	5.12 ± 0.29 ^c^	5.75 ± 0.47 ^bc^	6.66 ± 0.22 ^b^
NO (umol/gprot)	1.12 ± 0.03 ^d^	1.74 ± 0.15 ^c^	1.53 ± 0.03 ^c^	2.11 ± 0.17 ^b^	2.63 ± 0.12 ^a^
SOD (U/mgprot)	22.92 ± 1.46 ^a^	22.19 ± 0.44 ^ab^	19.85 ± 0.45 ^bc^	18.68 ± 0.23 ^c^	20.72 ± 0.07 ^abc^
MDA (nmol/mgprot)	21.24 ± 0.33 ^b^	25.56 ± 0.51 ^a^	22.57 ± 1.01 ^b^	23.04 ± 0.46 ^b^	22.25 ± 0.44 ^b^

The data were expressed as means ± SEM (n = 12). Different letters indicate significant differences (*p* < 0.05). T-AOC: total antioxidant capacity; TNOS: total nitric oxide synthase; iNOS: inducible nitric oxide synthase; NO: nitric oxide; SOD: superoxide dismutase; MDA: malondialdehyde.

## Data Availability

The original contributions presented in this study are included in the article. Further inquiries can be directed to the corresponding author.
